# Non-pathogenic microbiota accelerate age-related CpG Island methylation in colonic mucosa

**DOI:** 10.1080/15592294.2022.2160568

**Published:** 2022-12-26

**Authors:** Ang Sun, Pyounghwa Park, Lauren Cole, Himani Vaidya, Shinji Maegawa, Kelsey Keith, Gennaro Calendo, Jozef Madzo, Jaroslav Jelinek, Christian Jobin, Jean-Pierre J. Issa

**Affiliations:** a Fels Cancer Institute for Personalized Medicine, Temple University School of Medicine, Philadelphia, PA, United States; b Coriell Institute for Medical Research, Camden, NJ, United States; c Research Department of Pediatrics, University of Texas, MD Anderson Cancer Center Department of Pediatrics, University of Texas, MD Anderson Cancer Center Houston, TX, USA; d Department of Medicine, Division of Gastroenterology, Hepatology, and Nutrition, University of Florida College of Medicine, Gainesville, Florida, USA

**Keywords:** DNA methylation, Microbiota, Germ-free, Inflammation, Ageing

## Abstract

DNA methylation is an epigenetic process altered in cancer and ageing. Age-related methylation drift can be used to estimate lifespan and can be influenced by extrinsic factors such as diet. Here, we report that non-pathogenic microbiota accelerate age-related methylation drift in the colon when compared with germ-free mice. DNA methylation analyses showed that microbiota and IL10KO were associated with changes in 5% and 4.1% of CpG sites, while mice with both factors had 18% alterations. Microbiota, IL10KO, and their combination altered 0.4%, 0.4%, and 4% of CpG island methylation, respectively. These are comparable to what is seen in colon cancer. Ageing changes were accelerated in the IL10KO mice with microbiota, and the affected genes were more likely to be altered in colon cancer. Thus, the microbiota affect DNA methylation of the colon in patterns reminiscent of what is observed in ageing and colorectal cancer.

## Introduction

DNA methylation is an epigenetic mark with a profound impact on gene regulation and expression. This mark consists of the addition of a methyl group to a cytosine residue of a CG dinucleotide [1,2]. Approximately 80% of CpG sites in the human genome are methylated [3]. Many CpG sites are located in CpG islands (CGIs); these islands are 500–2000 bp stretches of DNA heavily enriched for C or G bases. They are generally unmethylated and are primarily located around transcription start sites (TSS). Methylation of TSS CGIs leads to gene silencing, but intergenic or gene body methylation can have varying effects, depending on the gene and the exact site that is methylated [4]. Disruption of DNA methylation patterns is associated with ageing and disease; this is characterized by global hypomethylation and aberrant hypermethylation of CGIs [1]. CGI hypermethylation leads to the down-regulation of key genes, including tumour suppressor genes, which can directly result in tumorigenesis [5,6]. Consequently, studying the causes of aberrant methylation is essential to our understanding of both ageing and cancer. Cell intrinsic factors such as the density of repetitive elements, baseline gene expression, and binding by PCG proteins can affect the propensity to aberrant DNA methylation in cancer [5, 7–10]. Cell extrinsic factors that modulate DNA methylation are less well defined, but they include ageing, diet, and chronic inflammation [1, 11–14].

The human gut microbiota is composed of approximately 10^13^ bacteria, which practically match the number of human cells in the body [15]. The gut microbiota has co-evolved with the host, forming a symbiotic relationship contributing to energy and nutrient extraction from diets, shaping immune response, maintaining intestinal mucosal barrier integrity, and performing key xenobiotic metabolism [16–20]. The gut microbiome is linked to inflammatory diseases, a major risk factor for cancer. In addition, the microbiome has been implicated in both ageing and inflammation. Germ-free (GF) mice raised and maintained without a microbiota have extended lifespans relative to their normal counterparts [21]. The microbiota has the ability to induce inflammation [22] and, in turn, inflammation has been shown to significantly alter microbiota composition [23].

Because of the common link to inflammation, there has been an interest in studying potential microbiota/epigenetic interactions. Microbiota have been shown to induce large-scale, diet-dependent changes in histone modifications [24]. A study of eight pregnant women examined the microbial composition and DNA methylation in the gut, finding that CGI methylation profiles differed based on microbial composition [25]. Additionally, infections have been linked to DNA methylation changes in cancer: Infection with *H. pylori* led to methylation of CGI promoters including TSGs in gastric cancer [26]. Also, in gastric cancer, a hypermethylation phenotype termed CpG Island Methylator Phenotype (CIMP) has been linked to Epstein–Barr virus (EBV) presence [27]. It was also shown that a specific family of bacteria, *Fusobacteria*, is markedly enriched in colorectal cancers (CRCs) with CIMP, but much less so in cancers without CIMP [28]. Based on these data, we hypothesized that the microbiota could induce changes in DNA methylation. To test this hypothesis directly, we compared DNA methylation in the colon of wild-type (WT) GF mice and WT mice inoculated with microbiota that were specific-pathogen-free (SPF). Further, we investigated the difference between the colonic DNA methylation of GF and SPF mice in a different genetic background, Interleukin 10 knockout (IL10KO), because IL10 is an anti-inflammatory cytokine and a previous study showed mice lacking the *IL10* gene (*IL10^−/−^*, IL10KO) develop spontaneous intestinal inflammation in the presence of microbiota [29]. We also studied interactions between the presence of microbiota and the DNA methylation of IL10 KO (*IL10^−/−^*) mice (prone to inflammation and tumorigenesis), ageing mice, and mice exposed to the carcinogen azoxymethane (AOM).

## Materials and methods

### Mouse colon samples

Mouse colon tissues for microbiota, IL10KO, and AOM studies were obtained from previously described experiments testing the effects of microbiota on colonic tumours [23]. IL10KO and WT SPF mice (129/SvEv) were born and raised in germ-free isolators and transferred to SPF facility at ages 7–12 weeks to acquire microbiota from the SPF environment for 20 weeks until sacrificed. Both the GF and SPF mice were born and raised in house at the Center for Gastrointestinal Biology and Disease Gnotobiotic Core animal facility at the University of North Carolina at Chapel Hill. Proximal colon tissues were obtained from the GF and SPF mice, which were sacrificed at approximately 8 months of age via C02 inhalation followed by cervical dislocation to ensure death (mice not anaesthetised prior to C02 exposure). Mouse colon tissues for ageing studies were obtained from six C57/B6 WT mice kept on regular diets and sacrificed at 4 (n = 3) or 30–33 (n = 3) months of age. Power analysis was performed for the ageing mouse model by calculating average of power for each CpG site with larger than 5% of methylation difference. The average power of the ageing mouse model is 0.90, and insured three mice is sufficient to detect the methylation variability between designed groups. All animal protocols were reviewed and approved by the Institutional Animal Care and Use Committee of the University of North Carolina at Chapel Hill and the Institutional Animal Care and Use Committee of Temple University.

We studied a total of 48 mice (Table 1).

**Table 1.** Type and number of mice studied for DNA methylation.

Mouse Model*	Number of Mice	Age at Collection (months)	Comment
WT/GF	6	8	Germ-free (GF) mice – no microbiota
WT/SPF	6	8	Mice with a Specific-Pathogen-Free (SPF) microbiota
IL10KO/GF	6	8	*Il10^−/−^* are predisposed to spontaneous inflammation but only in the presence of microbiota
IL10KO/SPF	6	8	
WT/SPF + AOM	6	8	Azoxymethane (AOM) is a colon carcinogen
IL10KO/GF + AOM	6	8	Predisposed to spontaneous inflammation and tumorigenesis but only in the presence of microbiota
IL10KO/SPF + AOM	6	8	
Young (C57/B6)	3	4	4 months old
Old (C57/B6)	3	30–33	30–33 months old

*All mice were 129/SvEv unless noted otherwise.

### Digital restriction enzyme analysis of methylation

Digital Restriction Enzyme Analysis of Methylation (DREAM) is a quantitative, deep-sequencing-based method of measuring DNA methylation at CpG sites within the CCCGGG sequence [30,31]. Genomic DNA (gDNA) was extracted from mouse proximal colon tissues as previously described [32]. Then, the gDNA was subjected to constructing libraries for next-generation sequencing following the protocol of performing DREAM published previously [30,31]. Briefly, mouse proximal colonic gDNA was sequentially treated with two restriction enzymes *Sma*I and *Xma*I, which act on the same DNA sequence but leave different 5’ ends of fragments. *Sma*I is blocked by CpG methylation, but *Xma*I is not. By treating gDNA first with *Sma*I, then *Xma*I, we create distinct signatures for unmethylated and methylated CpG sites at the edges of restriction fragments. Next, Illumina sequencing adapters were ligated to the ends of the restriction fragments to construct libraries, which were then sequenced by Illumina HiSeq 2500 instrument at the Fox Chase Cancer Center Genomics facility. We aligned the sequencing reads to the mouse genome (mm9) using Bowtie2, and counted signatures corresponding to unmethylated and methylated CpGs. There are 173,451 CCCGGG sites in mm9 genome assembly with 19,673 of them in CpG islands. At a minimum of 100 reads per CpG site, using DREAM, we could detect the methylation status of approximately 25,000 CpG sites on average. The sequencing results were filtered by a minimum sequencing depth of 100 reads on autosomes in at least 75% of the samples. We calculated methylation levels as the ratio of reads with the methylated signature to all reads mapping to each respective sites. The methylation values were then adjusted based on nine spiked-in standards, which have known methylation levels. At each *Sma*I/*Xma*I site, the *Xma* stood for the number of reads corresponding to methylated CpG (i.e., reads starting with CCGGG), and *Sma* was used as the number of reads corresponding to unmethylated CpG (i.e., reads starting with GGG). Then, the methylation value was calculated as 100% × (c × (Xma + 0.5)/(c × (Xma + 0.5) + Sma + 0.5)), where c was the correction factor calculated based on the observed and expected methylation of spiked-in standards. The correction factor characterizes the efficiency of enzyme digestion. We accepted data with correction factor values between 0.5 and 2.0 (1.0 means no correction).

### Bisulphite pyrosequencing for DNA methylation analysis

Bisulphite treatment of gDNA was performed by using the EpiTect Bisulphite Kit (Qiagen) following the manufacturer’s instructions. A quantitative bisulphite pyrosequencing method for DNA methylation analyses was used as previously reported [32–34]. Briefly, bisulphite-treated gDNA was amplified with gene-specific primers in a two-step PCR (Polymerase Chain Reaction). The reverse strand of DNA was labelled with biotin in the second step of the PCR. DNA methylation was quantified and indicated as the percentage of bisulphite-resistant cytosines at CpG sites by pyrosequencing performed following the instructions of the manufacturer using the PyroMark Gold Q96 CDT Reagents (Qiagen) on the PyroMark Q96 MD platform (Qiagen) and analysed by Pyro Q-CpG Software (Qiagen). For each biological sample, an average of three technical replicates were reported. The PCR primer sequences and conditions for bisulphite pyrosequencing of CpG sites in the promoter CGI of *Trhde*, a previously reported ageing-related gene [32], are listed in Supplementary Table 1.

### Quantitative RT-PCR analysis for gene expression

From the mouse proximal colon tissue samples, high-quality total RNA was extracted by using TRIzol (Invitrogen) following the manufacturer’s instructions. Then, complementary DNA was prepared by using a High Capacity cDNA Reverse Transcription Kit (Applied Biosystems) and followed the instructions from the manufacturer and used the total RNA as the template. The expression of *Trhde, Srrm4*, and *GAPDH* was quantified by a quantitative real-time polymerase chain reaction (qRT-PCR) performed by a StepOne Real-Time PCR system (Applied Biosystems) using TaqMan gene expression assays (Applied Biosystems).

### Pathway analysis

Pathway analysis was done using the R package ReactomePA, which is an R interface for pathway analysis using the open-source and manually curated Reactome pathway database (https://reactome.org/) [35]. Promoters were defined as the transcription start site, plus or minus 1500 bases. Promoters were considered changed if they had at least one significant differential methylation CpG site within them. For background, all promoters with at least one measured CpG were used. P-values were corrected for multiple testing using FDR.

### Multivariate linear regression model for the relationship between methylation and microbiota, IL10KO, and AOM treatment

For each CpG site, we generated a multivariate linear regression for the relationship between methylation and microbiota, Il-10 deficiency, and AOM treatment. The linear equation is defined by Equation [1] $y=b_{m}x_{m}+b_{i}x_{i}+b_{a}x_{a}+b_{0}+\varepsilon ,$ where y is methylation, x is the respective condition (m = microbiota, i = IL10KO, or a = azoxymethane respectively), b is the respective regression coefficient, and ε is the error term in predicting the value of Y.

### Statistical analyses

Statistical analyses were performed, and the figures were generated using R [36] (https://www.R-project.org/). Volcano plots were generated by plotting the average difference in methylation for a given CpG site against the negative log10 of the p-value. Average methylation change was calculated by subtracting average methylation in one condition from another, and an unpaired t-test was used to calculate a 2-tailed p-value. Bar plots were generated by taking the number of sites meeting a condition dividing by the number of sites examined. UpSet plots were generated using the ggupset package [37], while all other plots were generated using the ggplot2 package. All reported p-values are two-sided, and p ≤ 0.05 was used as a threshold of significance.

## Results

### Microbiota modulate DNA methylation

**Figure 1.**
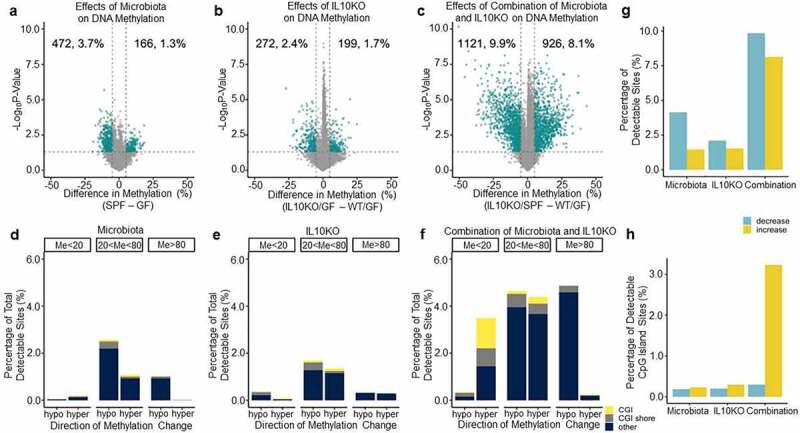
Microbiota influence DNA methylation. a-c) Volcano plot analysis showing methylation differences between SPF and GF mice (a), *Il10^−/−^* and GF mice (b), and SPF-*Il10^−/−^* and GF mice (c). The x-axis shows the difference in average methylation between SPF and GF mice for a given site. The y-axis is the negative log(10) of the p-value, which was determined with a Student’s t-test. All sites above the dotted line are significant at p ≤ 0.05. Green sites change at a magnitude of 5% or greater. d-f) Bar graphs showing the proportion and type of CpG sites that change at least 5% between SPF and GF mice (d), *Il10^−/−^* and GF mice (e), and SPF-*Il10^−/−^* and GF mice (f). Shore indicates sites that are not in CGIs but within 2,000 bp of them. (g) Bar graph showing the proportion of CpG sites that show DNA methylation alterations in the volcano plots in a-c. (h) Bar graph showing the proportion of CpG sites within CpG islands that show DNA methylation alterations in the volcano plots in a-c.

We used DREAM to examine how the presence of microbiota affects DNA methylation. To do this, DREAM data for WT GF and WT SPF mice were analysed and a volcano plot depicting the methylation differences between them was generated (Figure 1a). For most experiments, each group of mice consisted of six animals (Table 1). DREAM detected the methylation status of 24,865 sites on average at a minimum of 100 reads/site. We considered sites to be ‘changed’ if there was a statistically significant increase or decrease in average methylation of 5% or more. To ensure precision, we only analysed autosomal CpG sites that had greater than 100 reads in at least 75% of the mice in each group. Overall, of 12,919 detectable sites, 3.7% decreased, and 1.3% of sites increased in methylation. Thus, 5% of CpG sites analysed showed differences triggered by the presence of microbiota.

CpG sites that are greater than 80% or less than 20% methylated at baseline tend to be more stable than intermediately methylated sites [3]. Therefore, we examined how the microbiota affect different CpG compartments. Volcano plots were generated that looked only at sites with greater than 80% average methylation, sites with less than 20% methylation, and sites with between 20% and 80% methylation in GF mice (Supplementary Figure S1). Overall, 0.3% of unmethylated sites were affected by microbiota, compared to 1.1% of highly methylated sites, and 3.8% of intermediately methylated sites. Figure 1d shows the distribution of sites that were changed at least 5% between GF and SPF mice, stratified by baseline methylation. CpG island (CGI) and CGI shore sites appear to be largely stable. The most vulnerable sites seem to be gene body or intergenic areas with baseline methylation of between 20% and 80%. Thus, the presence of microbiota significantly affects DNA methylation in colonic mucosa with slightly more pronounced hypomethylation than hypermethylation.

### *Deletion of* Il10 *affects DNA methylation*

IL10 is an anti-inflammatory cytokine and mice lacking the gene develop spontaneous intestinal inflammation in the presence of microbiota [29]. To determine the effects of Il10 on colonic DNA methylation independent of microbiota, DREAM data from GF/WT and GF/IL10KO mice were compared; Figure 1b shows the volcano plot. Of all detectable sites, 2.4% decreased and 1.7% increased in methylation. Figure 1e shows the distribution of affected sites located in CGIs, CGI shores, or other sites; Like Figure 1d, Figure 1e shows that relatively fewer CGI/shore sites were subject to change, and most of the changes that did occur were at variable sites with 20% to 80% methylation (also see Supplementary Figure S1). GF/IL10KO mice have little detectable inflammation [23]; thus, the DNA methylation changes seen in these animals may be direct effects of Il10 deficiency or may be related to low grade/patchy inflammation that is not detectable using the usual assays.

*Il10* deficiency combined with microbiota results in inflammation and markedly accelerates colon tumorigenesis in mice [23,38]. By comparing DREAM data from GF/WT to SPF/IL10KO mice, we were able to examine the effects from the combination of IL10 deficiency and the presence of microbiota. In a differential methylation analysis (Figure 1c), we found that 8.1% of sites increased and 9.9% of sites decreased in average methylation. In addition to more sites changing average methylation, the combined effects of inflammation susceptibility and microbiota appear to be associated with a specific increase in methylation at CGIs and CGI shores. Figure 1f shows that 2% of all sites were CGI/CGI shore sites that increased in average methylation between WT/GF and IL10KO/SPF mice. This contrasted with the individual effects from IL10 deficiency or microbiota, which had little impact on CGIs or CGI shores. Figure 1g gives a broad overview of the effects of microbiota and IL10KO on DNA methylation individually and together. Both microbiota and IL10KO changed methylation at approximately 4–5% of sites. When combined, approximately 18% of sites experienced average methylation changes, with more sites decreasing in average methylation than increasing. When we examined CGIs exclusively (Figure 1h), 0.4% were changed by microbiota, 0.5% were changed by IL10KO while 3.5% were changed by the combination of microbiota and IL10-deficiency. These dramatic differences in DNA methylation are comparable to what can be seen when comparing cancer to normal [34].

### Differential effects of AOM and IL10KO on DNA methylation

**Figure 2.**
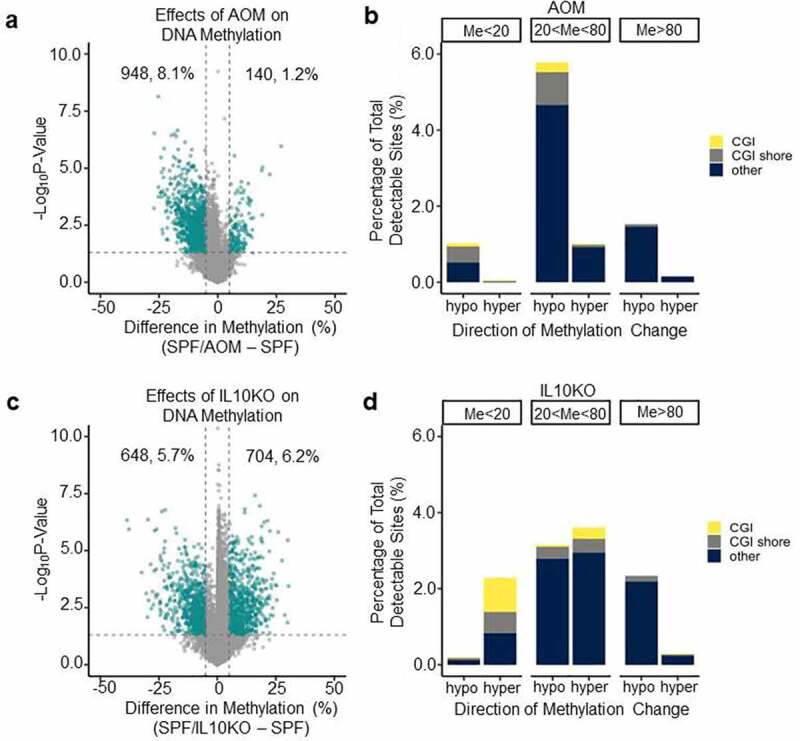
AOM is a potent hypomethylating carcinogen. (a) Volcano plot analysis showing methylation differences between SPF and SPF+AOM mice. See Figure 1 for graph details. (b) Bar graph showing the proportion and type of CpG sites that change at least 5% in (a). (c) Volcano plot analysis showing methylation differences between SPF and SPF-*Il10^−/−^* mice. See Figure 1 for graph details. (d) Bar graph showing the proportion and type of CpG sites that change at least 5% in (c).

AOM is a carcinogen commonly used to induce colorectal tumorigenesis in mouse models. We sought to determine if AOM and IL10KO differed in their effects on DNA methylation profiles. We examined the effects of AOM alone, IL10KO alone, and both in combination in the presence of microbiota (i.e., in SPF mice). A differential methylation analysis showed that AOM had a pronounced hypomethylating effect (Figure 2a). As a result of AOM treatment, approximately 8.1% of sites decreased in average methylation, as opposed to only 1.2% of sites increasing in average methylation. It is interesting to note that in both sites with 80% or more average methylation and 20% or less average methylation, AOM affected overall decreases in average methylation (Figure 2b). In addition, AOM did not seem to target CGI or CGI shore sites and was most likely associated with a decrease in methylation of sites with medium levels of baseline methylation (20% < Me < 80%, Figure 2b). By contrast, IL10KO in SPF mice led to 5.7% of sites decreasing in methylation and 6.2% of sites increasing in methylation (Figure 2c); about one-third of sites that increased in methylation were located in CGIs or CGI shores (Figure 2d). Thus, AOM and IL10KO both induced hypomethylation but, in the presence of microbiota, only IL10KO induced substantial CGI hypermethylation, pointing to potentially different mechanisms for their effects on DNA methylation.

### Shared and unique DNA methylation changes

**Figure 3.**
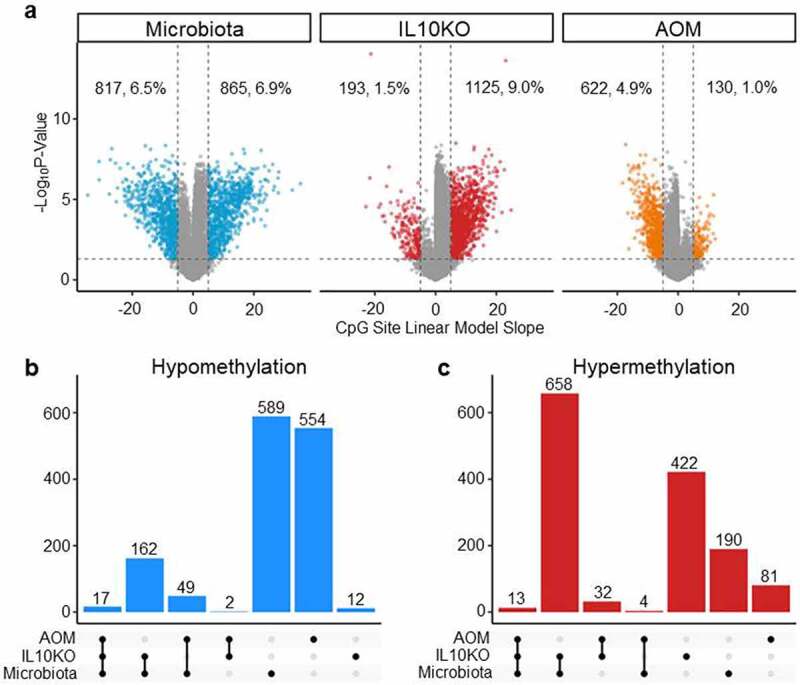
Linear regression model of the effects of microbiota, *Il10^−/−^*and AOM on DNA methylation. (a) Shows Volcano plots of FDR (0.05) corrected significant methylation changes attributed to each external exposure. The x-axis indicates the linear model slope for individual CpG sites (equivalent to % change in methylation) while the y-axis is the negative log(10) of the q-value. (b) Shows Upset plots of shared/unique hypomethylation events while (c) shows UpSet plots of shared/unique hypermethylation events. The number of shared or uniquely altered CpG sites is indicated on top of each vertical bar.

To elucidate the shared and unique effects of microbiota, *IL10* deficiency, and AOM treatment on DNA methylation, a multi-variate linear regression model that incorporates data from all 42 mice studied was built. Using a False Discovery Rate (FDR) of 0.05, we found that the linear model recapitulated the trends seen in the individual comparisons. Using this model (Figure 3a), we determined that microbiota (SPF) induced changes in 13.5% of CpG sites (6.9% hyper and 6.5% hypomethylated), IL10KO induced changes in 10.5% of sites (9% hyper and 1.5% hypomethylated), while AOM induced changes in 5.9% of sites (1% hyper and 4.9% hypomethylated). AOM continued to show a pronounced hypomethylating effect, while IL10KO continued to show a strong hypermethylating effect. Microbiota show roughly equal hypomethylating and hypermethylating effects. We used UpSet plots to determine the overlap in effects of the different factors on CpG methylation, examined separately for hypomethylation (Figure 3b) and hypermethylation (Figure 3c). Microbiota and IL10KO have the most shared events, particularly when it came to hypermethylation. Thus, of 865 sites hypermethylated by microbiota, 671 (78%) were also affected by IL10KO. There was less conservation when it came to hypomethylation (22% of sites affected by microbiota were also affected by IL10KO). AOM had mostly unique effects. These data suggest that microbiota and IL10KO have shared mechanisms of affecting DNA methylation, while AOM has an independent mechanism of action.

### The microbiota and inflammation accelerate age-related methylation drift

**Figure 4.**
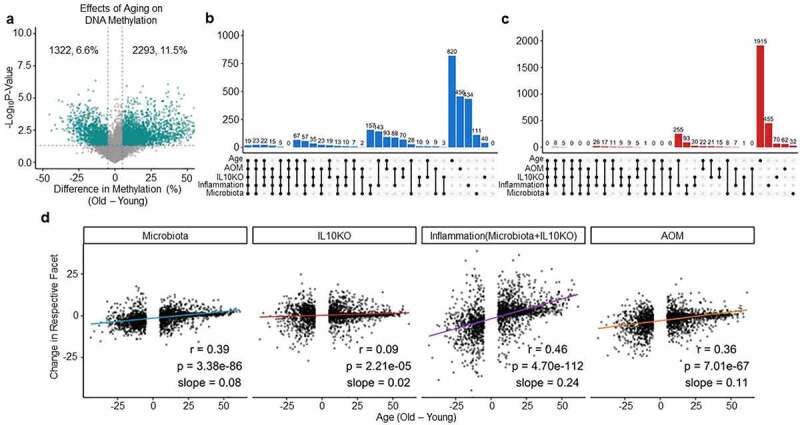
Microbiota and inflammation modify the same CpG sites subject to age-related methylation drift. (a) Volcano plot analysis showing methylation differences between young and old mice. See Figure 1a for graph details. (b) UpSet plots of shared/unique hypomethylation events between sites that decrease at least 5% during ageing and in the different exposures analysed in Figures 1–3. ‘Inflammation’ refers to *Il10^−/−^/*SPF mice. (c) Same analysis as in B for sites that increase methylation at least 5%. (d) Scatter plot of average methylation change with age (x-axis) to average change by exposures (y-axis) for all sites that change at least 5% with age. Pearson r, p-value, and slope are indicated in each plot.

Methylation drift characterized by a global loss of DNA methylation with simultaneous hypermethylation in CGI promoters occurs as a result of ageing [32,33]. To compare this to microbiota effects, we first generated DREAM data for old (aged 30–33 months) and young (aged 4 months) mouse colon (Figure 4a). A different strain was used for the ageing studies; however, a comparison between ageing and strain differential methylated regions showed little overlap, suggesting that it is exceedingly unlikely that the results presented in the paper can be explained by strain-specific effects (Supplementary Figure S6). Looking at ageing in C57/Bl6 WT mice, we found that of ~20,000 detectable CpG sites, 6.6% decreased in methylation, and 11.5% increased in methylation. Hypermethylated CGIs accounted for 7% of the changes. We analysed the overlap between sites that changed during methylation due to ageing and sites that changed due to microbiota, IL10KO, or AOM. Ageing had the largest effect individually, but there were many shared hypomethylation (Figure 4b) and hypermethylation (Figure 4c) events between ageing and the extrinsic exposures. For example, out of 472 sites hypomethylated upon microbiota exposure, 139 (29%) were also affected by age; of the 272 hypomethylated sites affected by IL10KO, 31% were also affected by age; of the 1121 sites hypomethylated in inflamed mice (IL10KO/SPF), 31% were hypomethylated as well in ageing mice. Similarly, out of 166 sites hypermethylated upon microbiota exposure, 18% were also affected by age; of the 199 sites affected by IL10KO, 33% were also affected by age; of the 926 sites hypermethylated in inflamed mice (IL10KO/SPF), 35% were also hypermethylated in ageing mice. This corroborates previous data demonstrating partial overlap between inflammation-related and age-related methylation [39], and implicates microbiota in this process.

One drawback of this analysis is the use of a threshold of 5% change in average methylation to consider a site affected by inflammation, microbiota, or ageing; this threshold could lead to underestimation of conservation because of sites that may change at a lower magnitude in one condition. To address this, we focused on the large number of sites that change with age and generated scatter plots comparing a site’s average change with age to change caused by exposures (Figure 4d). Interestingly, the correlation coefficients were positive and statistically significant for all exposures with the strongest correlations for inflammation (IL10KO/SPF, r = 0.46, *p* < 0.0001) followed by microbiota (r = 0.39, *p* < 0.0001), AOM exposure (r = 0.36, *p* < 0.0001), and IL10 deficiency (r = 0.09, *p* < 0.0001). Taken together, these data indicate that the vast majority of sites that change with ageing were affected by microbiota or inflammation (combination of microbiota and IL10KO). There were also sites affected by exposures but not by ageing (Supplementary Figure S2), most evident in mice with inflammation triggered by the combined effects of microbiota and IL10KO.

To get a sense of the functional impact of the genes which have concordant DNA methylation changes between ageing and microbiota in the logistic regression model, we performed pathway analysis on promoters containing at least one CpG site concordant between ageing and microbiota. We found that these promoters are enriched in 42 statistically significant pathways (*p* < 0.05, corrected by FDR), including 11 pathways with more than 10 promoters (Supplementary Figure S5). Interestingly, these include 4 that are related to the G-protein-coupled receptor (GPCR) pathway (GPCR ligand binding, G alpha (i) signalling events, signalling by GPCR, and GPCR downstream signalling). Overall, notable enriched pathways suggest potential effects on cell membrane proteins that respond to external signals and help regulate transcription, as well as proteins involved in cell–cell communication.

### Bisulphite and gene expression based validation

DREAM is based on differential restriction enzyme analysis and previously validated when compared to bisulphite-based data. To confirm this in the current data set, we randomly selected an affected promoter CpG island, *Trhde*, and studied it by bisulphite-pyrosequencing. As shown in Supplementary Figure S4, methylation of *Trhde* was lowest in WT-GF mice, and was increased by exposure to SPF, by Il10-KO, by the combination of the two, but not by AOM treatment. These data closely mirrored what we observed by DREAM analysis. We next examined *Trhde* gene expression by qRT-PCR. We had limited tissues available for gene expression analysis but were able to demonstrate that *Trhde* is repressed in the presence of microbiota (Supplementary Figure S3), consistent with the finding of increased promoter CpG island methylation. As a control, we examined Srrm4, a gene unaffected by DNA methylation, and found no difference in gene expression (Supplementary Figure S3).

### CpG sites affected by microbiota are hypermethylated in colon cancer

**Figure 5.**
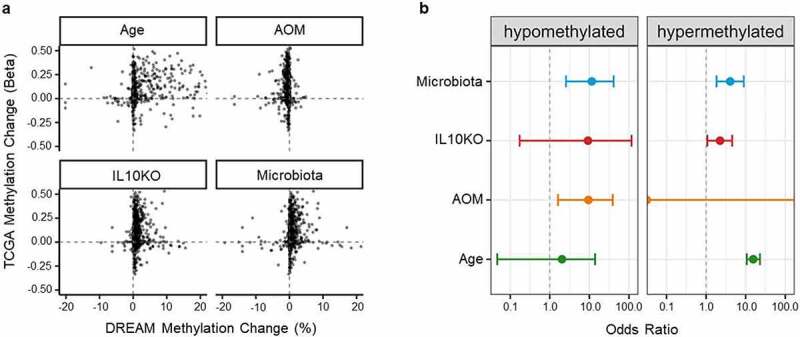
Genes affected by extrinsic exposures are more likely to be altered in cancer. (a) Scatter plot of DNA methylation change by ageing or exposure (x-axis) compared to DNA methylation change in colon cancer TCGA samples. (b) Odds ratios compute enrichment of genes altered by ageing or different extrinsic exposures among genes altered in colon cancer (TCGA data). The odds ratios are computed separately for gene promoters hypomethylated (left) or hypermethylated (right) by exposures and in colon cancer.

To determine the impact of microbiota, inflammation, and/or ageing on methylation in colon cancer, we analysed data from The Cancer Genome Atlas (TCGA). To correspond mouse to human data, we focused on promoters (defined as −1500 to 500 of transcription start site) and used data on the CpG site closest to the transcription start site. Converging DREAM data with TCGA data, we were able to analyse 2,042 genes in total. Figure 5a shows scatter plots of methylation changes in colon cancer patients included in the TCGA data set (β value of methylation difference between tumour and normal) plotted on the y-axis, and methylation changes in our datasets (based on the volcano plot for ageing and on the linear model for individual factors) plotted on the x-axis. The plots demonstrate a strong concordance between methylation changes in TCGA and each of ageing, microbiota exposure, and *IL10* deficiency. We calculated odds ratios to quantitate this concordance, at a threshold of 5% change in TCGA and in the risk factors (Figure 5b). For hypermethylation, the odds ratios for enrichment were 15.5 (95% CI: [10.6,22.9]; q = 3.0 × 10^−54^) for age, 0 (95% CI: [0,0]; q = 1) for azoxymethane, 2.3 (95% CI: [1.1,4.5]; q = 0.039) for IL10KO, and 4.1 (95% CI: [1.8,9.0]; q = 9.4 × 10^−4^) for microbiota. For hypomethylation, odds ratios were 2.0 (95% CI: [0.04,13.9]; q = 0.47) for age, 9.4 (95% CI: [1.6,38.9]; q = 0.015) for azoxymethane, 9.2 (95% CI: [0.2,116.2]; q = 0.18) for IL10KO and 11.5 (95% CI: [2.6,41.1]; q = 0.003) for microbiota. Thus, as previously reported, ageing has a major impact on whether a gene becomes hypermethylated in cancer, but we also find that genes affected by microbiota and by Il10 deficiency are also overrepresented among genes hypermethylated in CRC.

## Discussion

We show here that the presence of a microbiota is associated with changes in DNA methylation that affect 5% of the detectable CpG sites, a high number when one considers the fact that DNA methylation is very carefully controlled with relatively low levels of inter-individual variability [3]. These effects are compounded by the presence of inflammation – mice with both IL10 deficiency and exposure to a microbiota showed alterations in 18% of the detectable CpG sites, and the differences were particularly striking at CpG islands, regions that are normally very stable and highly protected from DNA methylation [40]. This strikingly high number is quantitatively similar to what can be seen when comparing mice at the extremes of their lifespan [34], and the pattern of change is very similar to what can be seen in colon cancers (CGI hypermethylation, intergenic hypomethylation). Indeed, CpG sites affected by microbiota are overrepresented among genes hypermethylated in colon cancer, suggesting that microbiota could have an important influence on shaping cancer epigenomes, as previously suggested [28]. Interestingly, while the presence of microbiota accelerates age-related methylation drift, there are also epigenetic changes specific to the different extrinsic factors studied (microbiota, inflammation, AOM) suggesting distinct mechanisms rather than non-specific effects of cycles of injury and stem cell proliferation. It is also worth noting that the effects we observe are related to pathogen-free microbiota. It would be interesting to determine whether more profound changes are seen when pathogenic bacteria are introduced into the mix.

Our results are consistent with recent studies but extend the findings substantially. Three studies reported that microbiota affect the physiology and DNA methylation patterns in developing intestinal epithelium [41–43]. Interestingly, the effects seen were primarily outside promoters (e.g., 3’ end of genes and inter-genic areas), while our results highlight the profound effect of microbiota and inflammation on promoter CpG islands – the compartment most uniquely affected in ageing and cancer [1]. Ansari also studied the effects of inflammation induced by Dextran Sodium Sulphate on DNA methylation, and found primarily hypomethylation [43], in marked contrast to what we observed with inflammation induced by the combination of IL10 deficiency and the presence of microbiota. Dextran Sodium Sulphate’s effects are reminiscent of what we observed with exposure to azoxymethane and raise the possibility that the effects seen are related to the chemical’s effect more directly than to inflammation. The effect of DNA damage by azoxymethane on DNA methylation is an important possibility. Increased yH2AX^+^ nuclear foci were observed in AOM/*Il10*^−/−^ mice with *E. coli* NC101 in colon [23]; however, we do not know if DNA damage induced by AOM per se is contributing to the altered methylation patterns we are observing. Finally, Sobhani et al. introduced into germ-free mice human faecal microbiota from patients with colon cancer or controls and also observed alterations in DNA methylation [44], and some of the genes affected (such as *SFRP1*) are known to show age-related methylation in the human colon [45]. In our study, we show that even pathogen-free microbiota affect DNA methylation at relatively high levels. Our data uniquely highlight the effects of microbiota and inflammation on aberrant CpG island DNA methylation and link it to the acceleration of ageing effects on DNA methylation. The data also suggest that microbiota/inflammation effects are potential precursors to the aberrant DNA methylation seen in colorectal cancers.

The mechanism by which microbiota affect DNA methylation remains to be elucidated. One possibility is via the production of metabolites which act as cofactors or inhibitors for epigenetic enzymes. For example, the microbial metabolites acetate, propionate, and butyrate are capable of inducing mass reprogramming of histone methylation and acetylation states [24]. Bacterial species producing these short-chain fatty acid metabolites are known to be decreased in cancer and inflammation. DNA methyltransferases (DNMTs) have been shown to interact with modifications on histone tails [46], and as histone states change, DNA methylation patterns may as well. Additionally, the gut bacteria may cause alterations in host gene expression that affect the ability of host cells to uptake certain metabolites; for example, it has been demonstrated that *E. coli* is capable of downregulating a protein responsible for butyrate uptake [47]. Aberrant DNA methylation may also be directly initiated by the overproduction of oncogenic metabolites (oncometabolites). Loss-of-function mutations in citric acid cycle enzymes fumarate hydratase (FH) and succinate dehydrogenase (SDH) result in the accumulation of fumarate and succinate, respectively, [48]. A neomorphic mutation in isocitrate dehydrogenase (IDH) causes the enzyme to produce 2-hydroxyglutarate (2HG) instead of α-ketoglutarate [49]. Each of these mutations is found in certain types of CIMP positive cancer [50], though they are very rarely observed in colorectal cancer [51]. 2HG binds to and inhibits α-ketoglutarate dependent TET and methyltransferase enzymes, causing DNA hypermethylation and tumorigenesis [50]. Succinate and fumarate have been shown to inhibit these enzymes *in vitro* and there is overlap between the methylated genes associated with each metabolite anomaly, implying that all three may act via this same mechanism [48]. Thus, it is plausible that bacteria cause aberrant methylation in part via over-production of metabolites that modulate the function of the TET DNA demethylases.

Diet is important to note for its effect on the methylation drift in colorectal cancer and ageing [13,32] and closely associated with the composition of the gut microbiota and the production of the bacterial metabolites mentioned above [52]. Introducing diet as another variable in the study design would be an interesting subject to explore in the future studies.

Previous studies have also shown that inflammation alters the DNA methylome [1,53], though the precise mechanisms by which this occurs are unknown. It has been proposed that inflammation increases cell turnover rate, and inflammation-induced methylation may simply be a reflection of an increased rate of age-related methylation [13]. The overlap we observed between inflammation and ageing-associated changes is consistent with this. However, we found that microbiota and inflammation also induced methylation changes that were not observed in ageing. It has been suggested that direct interactions with inflammatory cytokines alter a cell’s methylation profile [54]. It is also possible that the inflamed gut exerts different selective pressures on the host intestinal epithelial cells than the non-inflamed gut, selecting for cells with unique DNA methylation profiles. It may eventually be possible to tease out inflammation-independent effects of extrinsic factors on DNA methylation.

In conclusion, we find that microbiota have a profound effect on DNA methylation in the gut and help shape the aberrant methylomes seen in ageing and cancer. The fact that extrinsic factors (bacteria, inflammation, carcinogens) modulate the epigenome suggests potential ways to intervene therapeutically for cancer prevention.

## Supplementary Material

Supplemental Material

## Data Availability

The datasets supporting the conclusions of this article are available in the GEO (accession number: GSE150333, https://www.ncbi.nlm.nih.gov/geo/query/acc.cgi?acc=GSE150333). A secure token (kjizmsauldorlcn) has been created to allow editors and reviewers to review record GSE150333 while it remains in private status. Further information and requests for resources and reagents should be directed to and will be fulfilled by the Lead Contact, Jean-Pierre J. Issa.
