# Supplementary material for: Non-pathogenic microbiota accelerate age-related CpG Island methylation in colonic mucosa
Source: Epigenetics. 2022 Dec 26;18(1):2160568. doi: 10.1080/15592294.2022.2160568 (PMC9980687; doi:10.1080/15592294.2022.2160568)
Supplement: Supplemental Material [file KEPI_A_2160568_SM9629.zip › supplement/Supplementary Figures and Legends.docx]

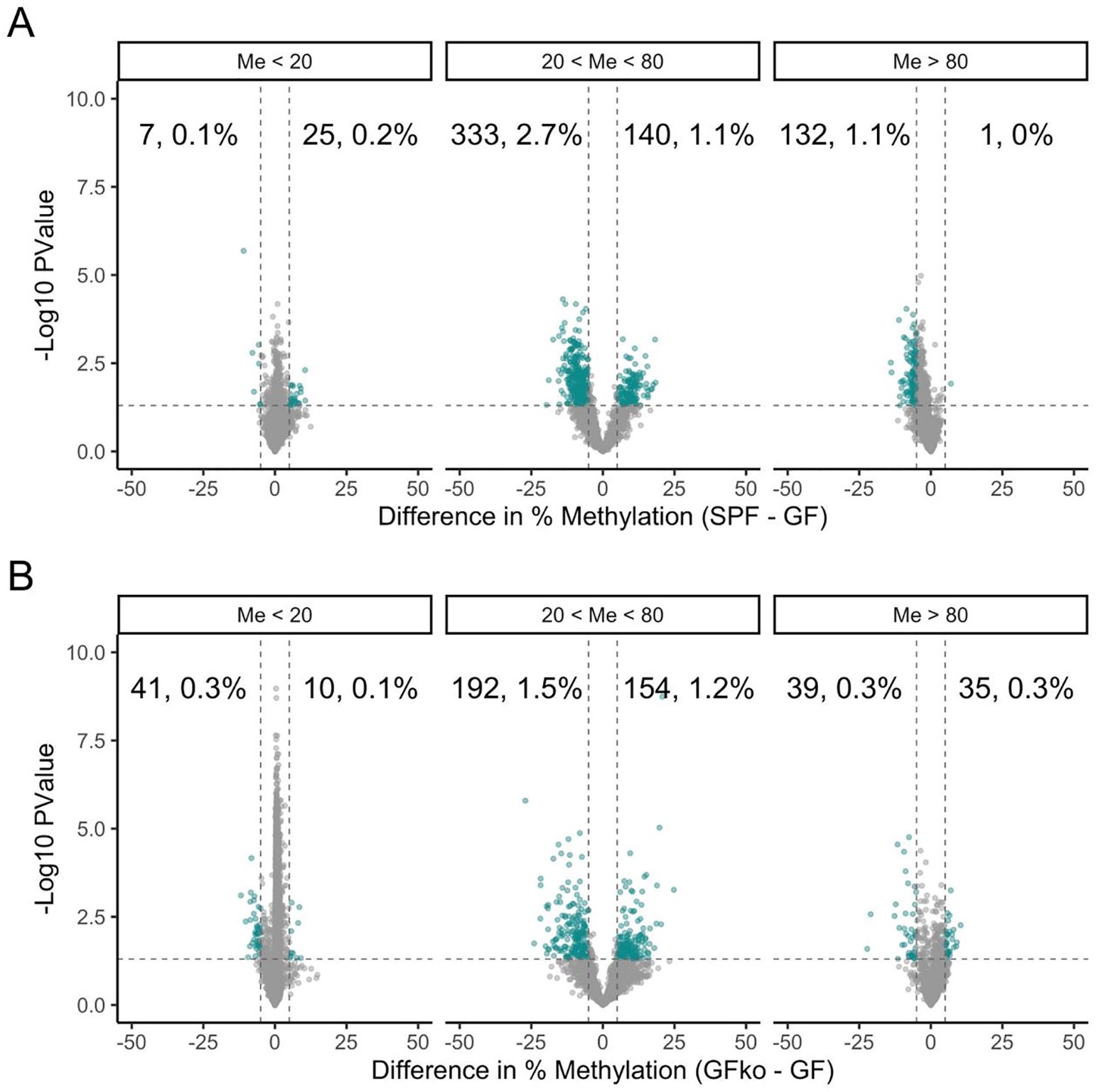


**A** ***

**B** ***

**C** ***

N.S.

**D**

**E** ***

**F** ***

1. N.S.


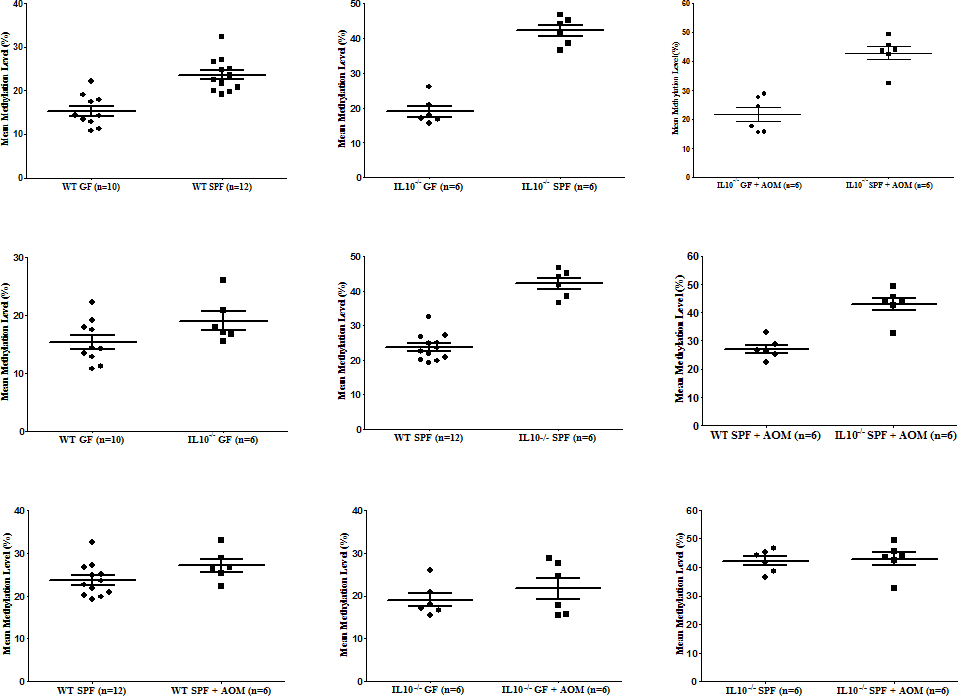


1. N.S.
2. N.S.


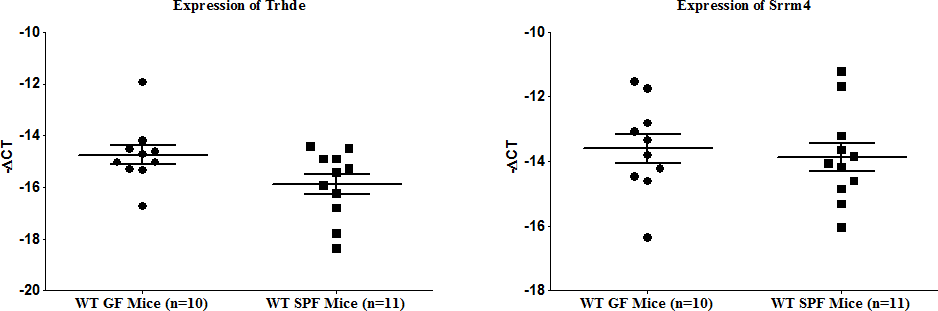


A

B

P=0.0536

N.S. P=0.6556

#
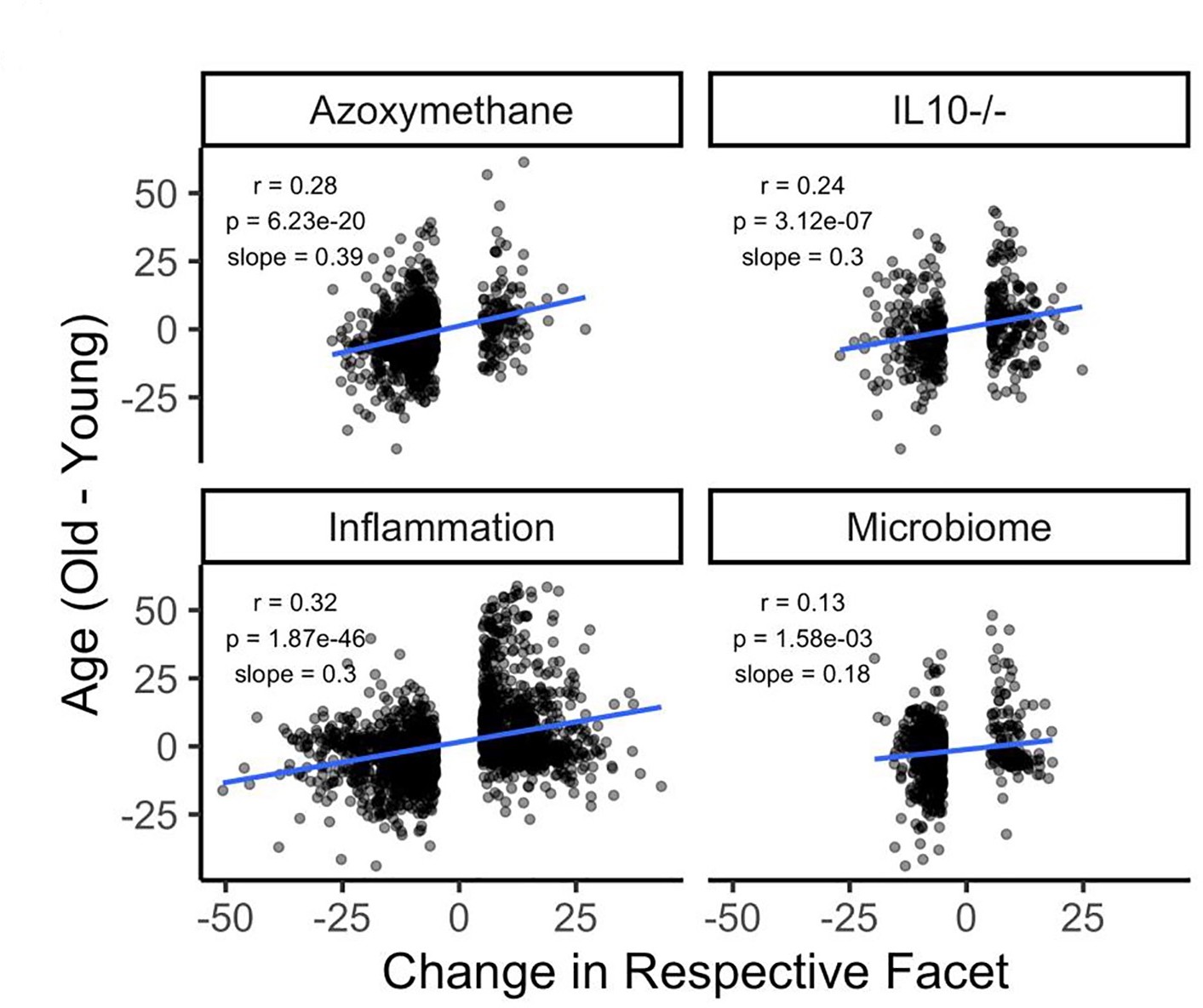
Figure S4.


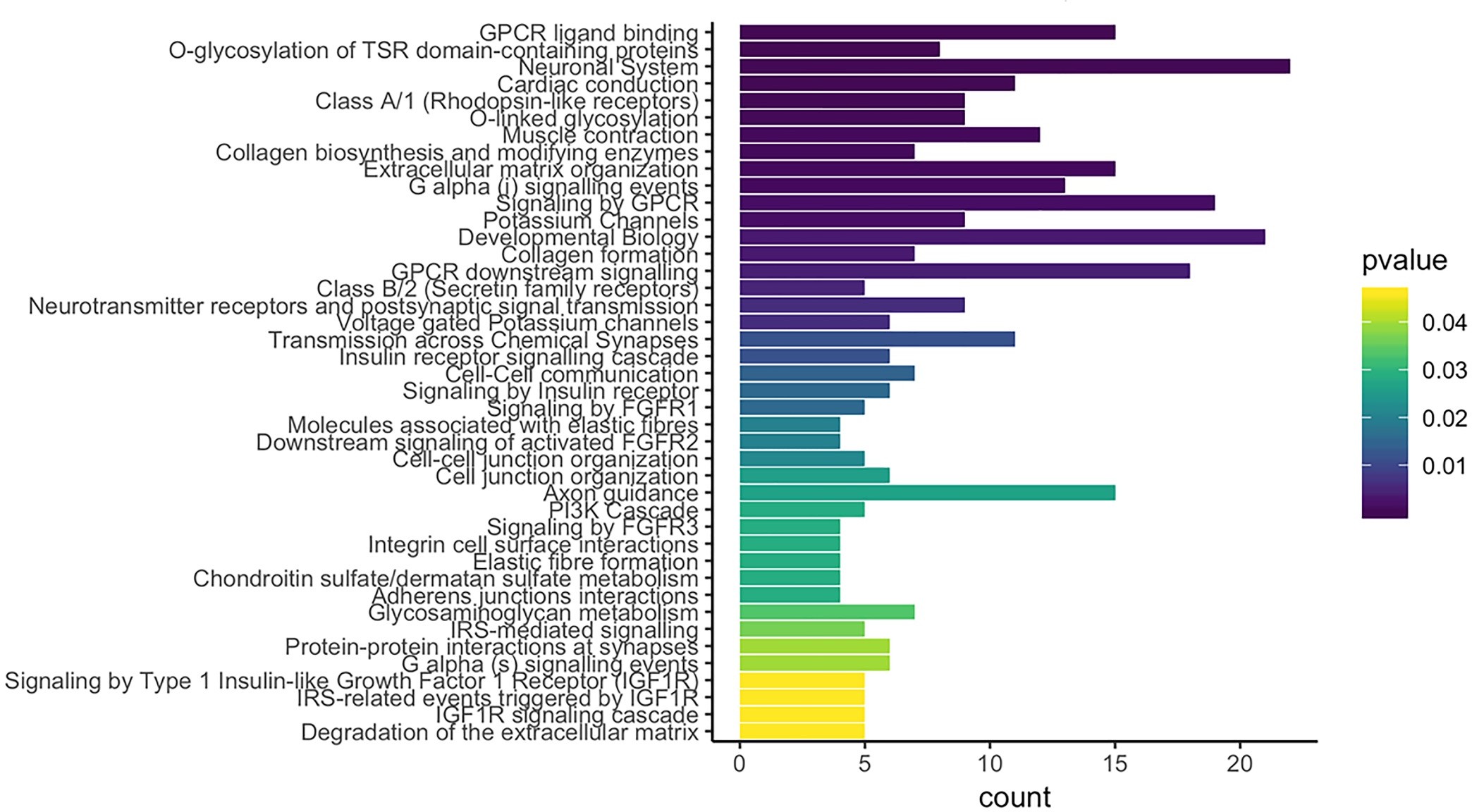
Enriched Pathways for Promoters Concordant between Aging and Microbiota


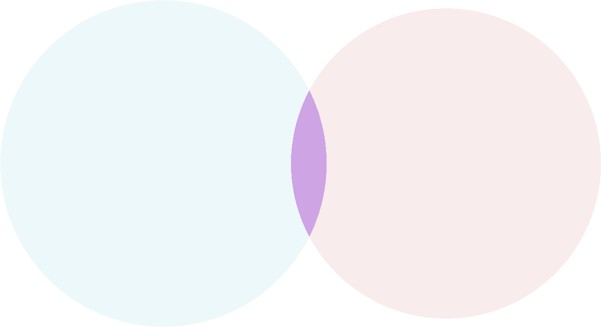


15402

676

13829


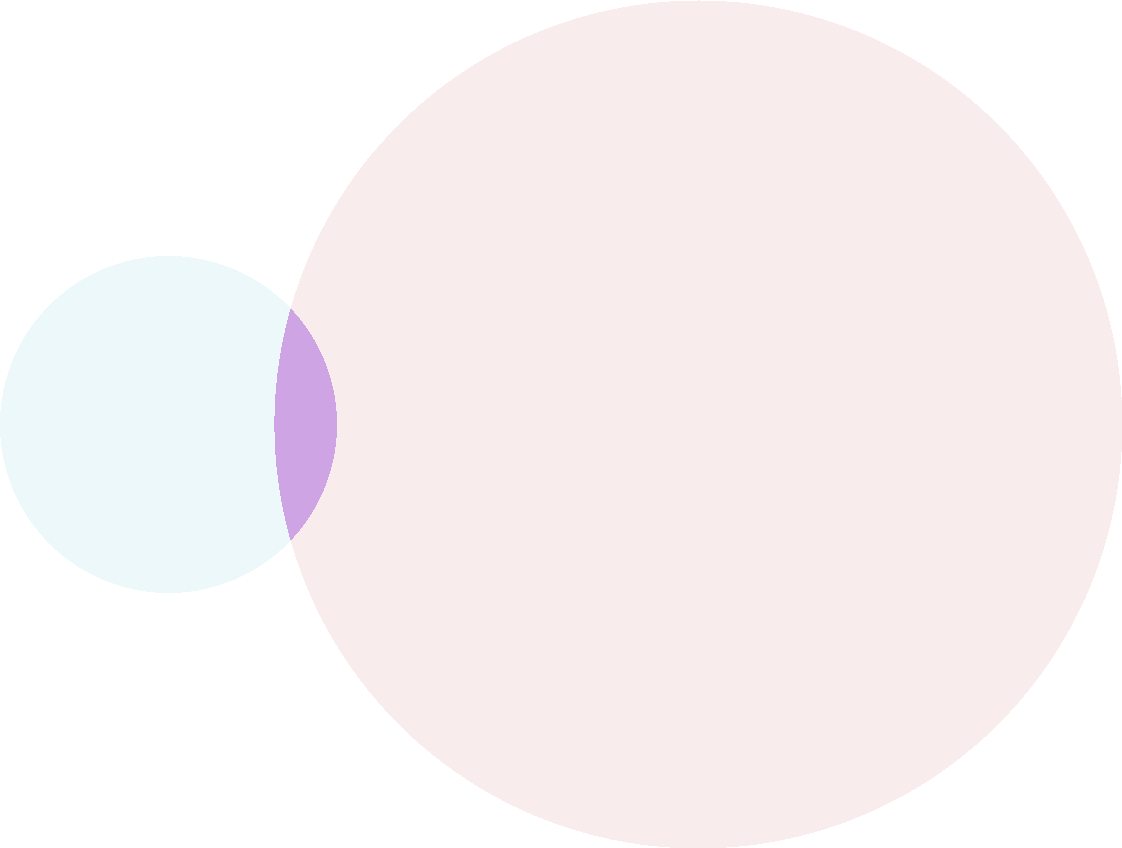

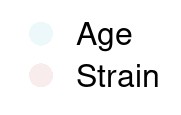


A

B

3981 499

27860


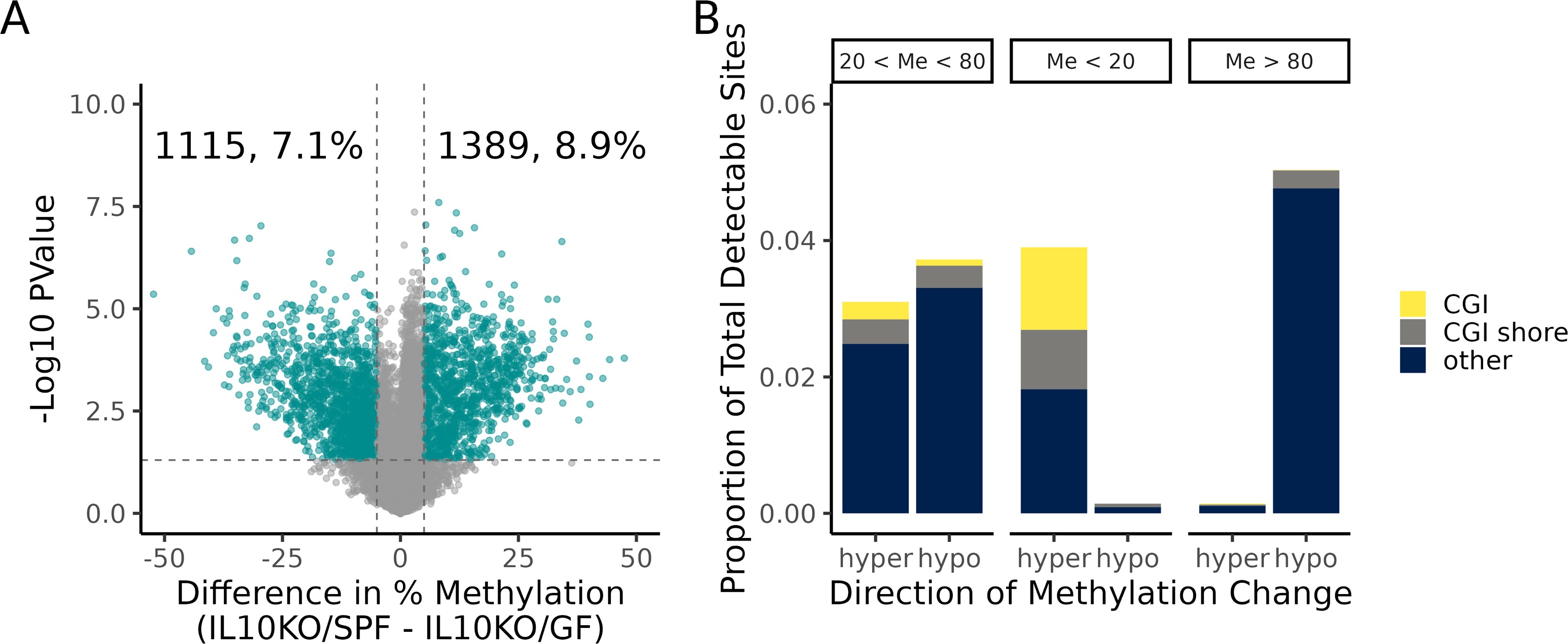


# C


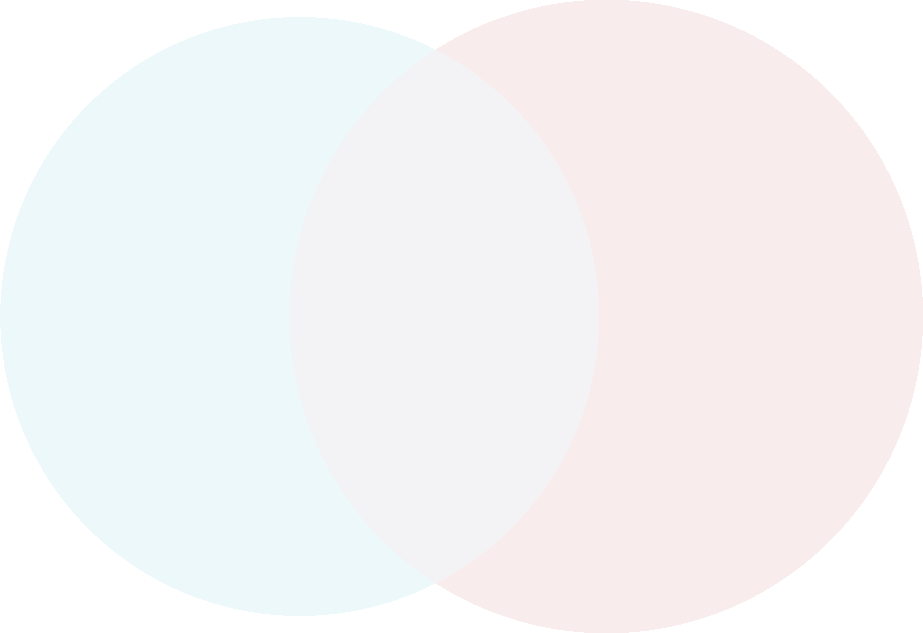

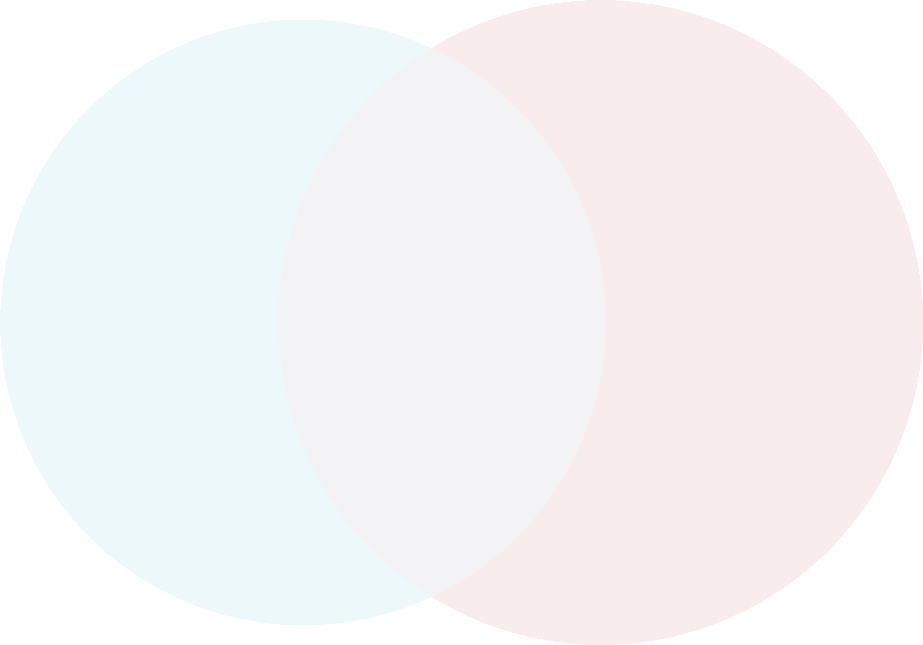

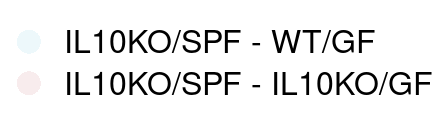


D

1121

897

1389

926

661

1115


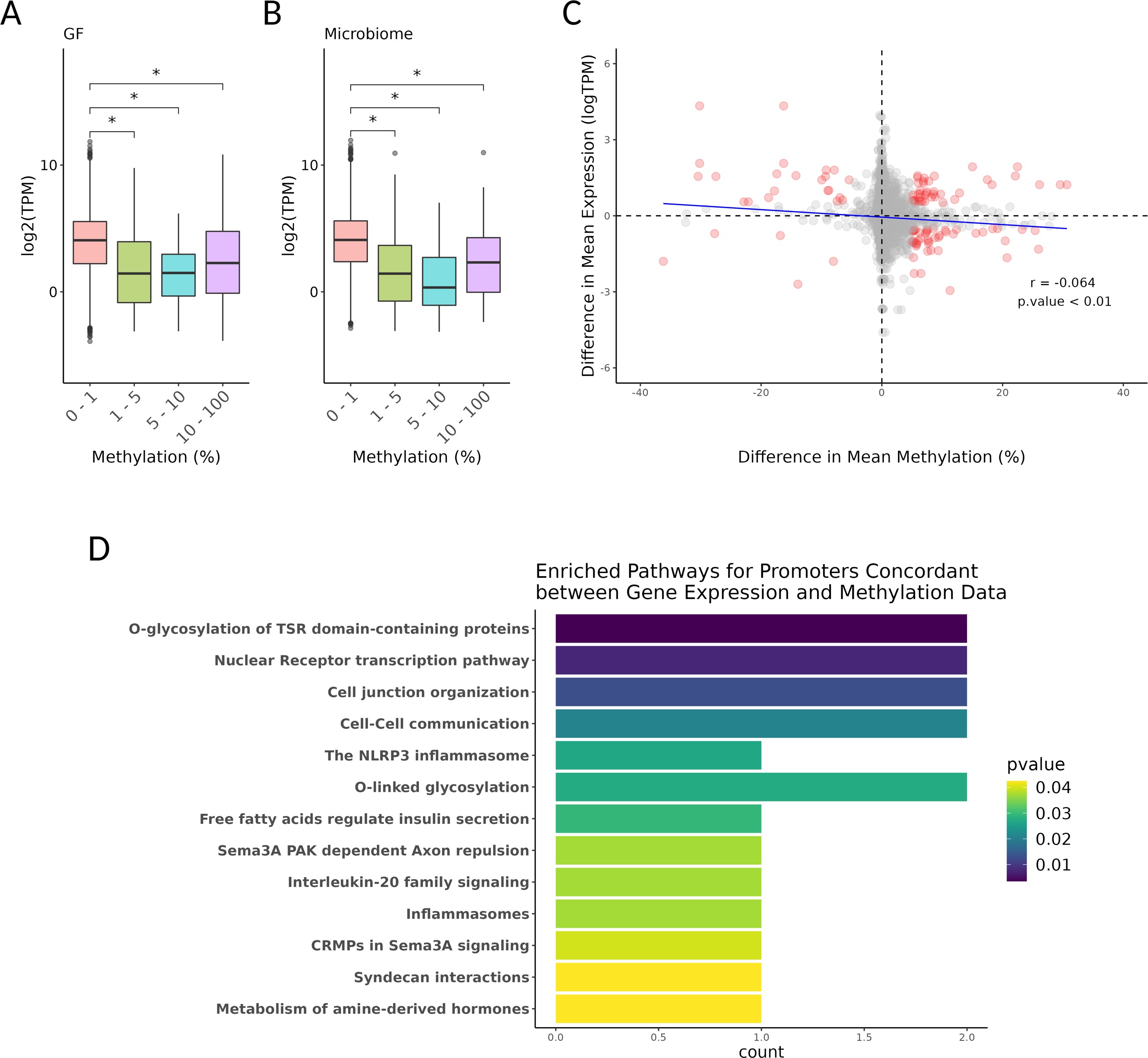


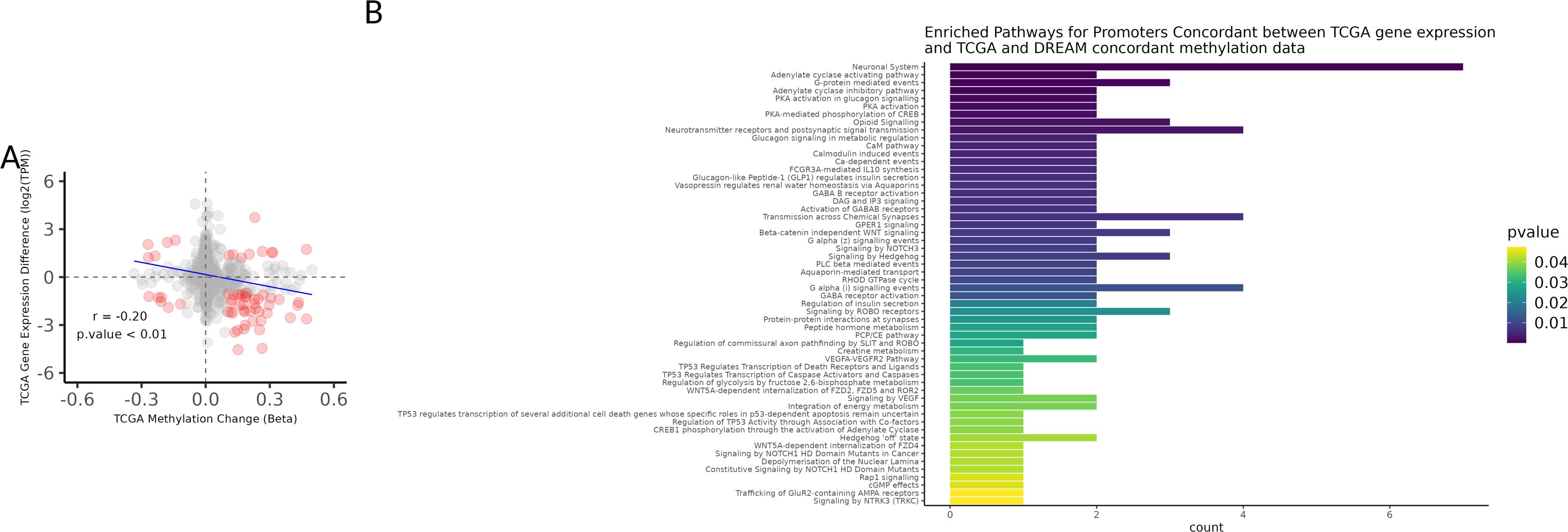


**Supplementary Figure Legends:**

**Figure S1:** Microbiota influences DNA methylation, stratified by baseline methylation. (**a**) Volcano plots show methylation differences between GF and SPF mice, analyzing sites with less than 20% methylation on average (left), sites with between 20% and 80% methylation (middle), and sites with greater than 80% methylation (right). (**b**) Similar analyses of methylation differences between *Il10-/-* and GF mice.

**Figure S2:** Bisulfite pyrosequencing of CpG sites within the prompter CGI of an aging-related gene, *Trhde*, between different groups of mice. The graphs demonstrate the effects of non-pathogenic microbes (A-C), *IL10* deficiency (**d-f**) and AOM 1Treatment (**g-i**) on DNA methylation. *** Indicate p<0.05.

**Figure S3:** Gene expression of *Trhde* and *Srrm4* was quantified by qRT-PCR. The expression of both

*Trhde* and *Srrm4* was compared with the expression of an endogenous control, *GAPDH*, and indicated by

–ΔCT. The greater the –ΔCT indicates the higher the gene expression level.

(**a**) *Trhde* is both an aging-related gene and a non-pathogenic microbiota-affected gene. Its expression was compared between WT GF mice (n=10) with WT SPF mice (n=11). (**b**) *Srrm4* is not an aging-related gene, nor has it been observed with higher DNA methylation level of its promoter region in WT SPF mice than WT GF mice. It is a gene randomly picked as a negative control. Its expression was compared between WT GF mice (n=10) with WT SPF mice (n=11).

**Figure S4:** Microbiota and inflammation modify the same CpG sites subject to age-related methylation drift. Shown are scatterplots similar to Figure 4(**d**) but with reversed axes, with average methylation change with age (y-axis) to average change by exposures (x-axis) for all sites that change at least 5% with exposures. Pearson R, p-value, and slope are indicated in each plot.

**Figure S5:** Enriched pathways for promoters concordant between aging and microbiota. Promoters with at least one detectable CpG site concordant between aging and microbiota from the logistic regression model were analyzed by using the R package ReactomePA to show the enriched pathways. There are 42

enriched pathways color-coded by FDR corrected p-value (p<0.05) with the count number of promoters within each pathway shown on the x-axis.

**Figure S6**: Aging-specific and strain-specific CpG sites comparison. a-b) Venn diagrams showings shared and unique significantly hypomethylated CpG sites (a), hypermethylated CpG sites (b) in aging and strain comparisons.

**Figure S7**: Microbiota influence DNA methylation. (a) Volcano plot analysis showing methylation differences between IL10KO/SPF and IL10KO/GF mice. The x-axis shows the difference in average methylation between SPF and GF mice for a given site. The y-axis is the negative log(10) of the p-value, which was determined with a Student’s t-test. All sites above the dotted line are significant at p-value ≤

0.05. Green sites change at a magnitude of 5% or greater. (b) Bar graphs showing the proportion and type of CpG sites that change at least 5% between IL10KO/SPF and IL10KO/GF mice. (c-d) Venn diagrams showing shared and unique significantly hypomethylated CpG sites (c), hypermethylated CpG sites (d) between two groups.

**Figure S8**: Association between DNA methylation and gene expression in different microbiota groups. (a- b) Bar plots showing the relationship between baseline DNA methylation on the x-axis and gene expression on the y-axis in the GF mice (a) and the mice with microbiome (b). (c) Scatter plot showing the relationship between the difference in mean methylation (x-axis) and the difference in mean expression (y-axis) of the common promoters between the gene expression and the methylation data. (d) Enriched pathways for promoters concordant between gene expression and methylation data.

**Figure S9**: Comparison between TCGA colorectal cancer gene expression and TCGA methylation data concordant with the mouse methylation data. (a) Scatter plot showing the relationship between methylation change (beta value) on the x-axis and gene expression (log2(TPM)) on the y-axis. (b) Enriched pathways for promoters concordant between TCGA gene expression and TCGA methylation data.
